# Dense breasts and women's health: which screenings are essential?

**DOI:** 10.1016/j.clinsp.2025.100743

**Published:** 2025-08-09

**Authors:** Bruna Salani Mota, Carlos Shimizu, Yedda N. Reis, Rodrigo Gonçalves, Jose Maria Soares Junior, Edmund Chada Baracat, José Roberto Filassi

**Affiliations:** Faculdade de Medicina, Universidade de São Paulo (USP), São Paulo, SP, Brasil

**Keywords:** Dense breast, Breast cancer screening, MRI

## Abstract

This review synthesizes current evidence regarding optimal breast cancer screening strategies for women with dense breasts, a population at increased risk due to decreased mammographic sensitivity. A systematic literature review was performed in accordance with PRISMA criteria, covering MEDLINE, EMBASE, CINAHL Plus, Scopus, and Web of Science until May 2025. The analysis examines advanced imaging techniques such as digital breast tomosynthesis (DBT), contrast-enhanced spectral mammography (CESM), ultrasound, and magnetic resonance imaging (MRI), assessing their effectiveness in addressing the shortcomings of traditional mammography in dense breast tissue. The review rigorously evaluates the incorporation of risk stratification models, such as the BCSC, in customizing screening regimens, in conjunction with innovative technologies like liquid biopsy and artificial intelligence-based image analysis for improved risk prediction. A key emphasis is placed on the heterogeneity in international screening guidelines and the challenges in translating research findings to diverse clinical settings, particularly in resource-constrained environments. The discussion includes ethical implications regarding compulsory breast density notification and the possibility of intensifying disparities in health care. The review ultimately encourages the development of evidence-based, context-specific guidelines that facilitate equitable access to effective breast cancer screening for all women with dense breasts.

## Highlights

• Mammographic density impacts the primary screening process, as dense tissue may mask tumors, resulting in false-negative results.
• Mammographic density is known to be a risk factor for breast cancer, associated with a 1- to 6-fold increase in the incidence of this disease.
• In women at average risk, going to annual screening mammography at age 40 has demonstrated a reduction in breast cancer mortality by roughly 20 %.
• For women at intermediate and high-risk, supplementary modalities such as magnetic resonance imaging, ultrasound, tomosynthesis, and molecular breast imaging may be considered for enhanced screening to increase detection rates.

## Introduction

Dense breasts constitute a challenge in the clinical practice of gynecologists. This arises from two flaws: dense breasts correlate with lower mammography sensitivity and increasing interval cancer rates^1^, ^2^, ^3^, ^4^, ^5^, as well as an elevated breast cancer risk of 1 to 6 times only due to the presence of dense breast tissue^1^^,^^6^

## Methods

A systematic review of the literature synthesizing findings from searches of electronic databases, including MEDLINE (PubMed), EMBASE, CINAHL Plus, Scopus, and Web of Science, was conducted until May 2025 in accordance with PRISMA guidelines using the Mesh terms “Dense breast” and “screening”. Data from the identified materials were extracted and presented in a narrative summary.

### What defines breast density, and how is it classified?

**Fig. 1:**
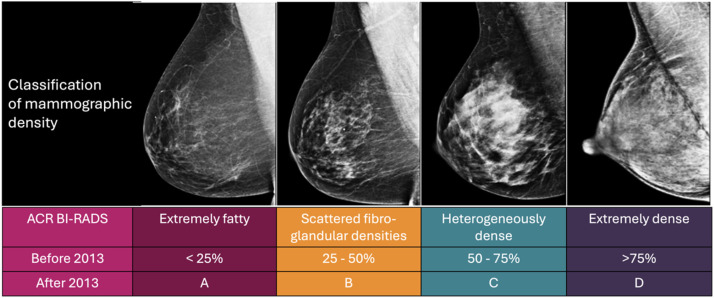
Classification of mammographic density.

Dense breast tissue indicates the white radiographic appearance of fibroglandular tissue detected by mammography. It is quantified based on the ratio of radiologically dense (white) regions to black portions (adipose tissue)^7^ Since its conception in 2013, the Breast Imaging Reporting and Data Systems 5th edition (BI-RADS) has emerged as the most widely used tool for the clinical classification of mammographic density^8^ The specified approach classifies breast density into four categories: extremely fatty (A), scattered fibroglandular densities (B), heterogeneously dense (C), and extremely dense (D). This classification is subjective and is based on the BI-RADS 4th edition utilized until 2013, wherein extremely fatty breasts consist of up to 25 % breast tissue; sparse fibroglandular tissue comprises 25 %‒50 %; heterogeneously dense tissue ranges from 50 %‒75 %; and extremely dense breasts have ≥ 75 % glandular tissue, as illustrated in Fig. 1. Breasts categorized as C and D are considered dense breasts.

To reduce the subjectivity in mammographic density classification, there has been an increase in the development of automated techniques, particularly Convolutional Neural Network (CNN) models, which demonstrate a high level of concordance with multiple radiologists. This progress aims to standardize breast density assessments and establish a reliable method for large-scale screening, giving promising outcomes^9^, ^10^, ^11^

### Prevalence of mammographic densities

Approximately 43 % of women aged 40 and 74 have breasts that are either extremely dense or heterogeneous (types C and D). According to the findings of an analysis of 1518,599 mammogram records from 764,507 women aged 40 and older, the percentage of mammograms that were classified as heterogeneous or extremely dense decreased with increasing age. This percentage decreased from 56.6 % (95 % CI 56.1 % to 57.0 %) for women aged 40 to 44 years to 28.4 % (95 % CI 27.4 % to 29.5 %) for women aged 85 and older. The percentage of women who had extremely dense breasts decreased from 12.5 % (95 % CI 12.4 % to 12.7 %) in women aged 40 to 44 to 3.1 % (95 % CI 2.8 % to 3.4 %) in women aged 85 and older, with an average prevalence of 7.4 % (95 % CI 7.4 % to 7.5 %) among women aged 40 to 74 years^12^

### Mammographic density and histopathology

**Fig. 2:**
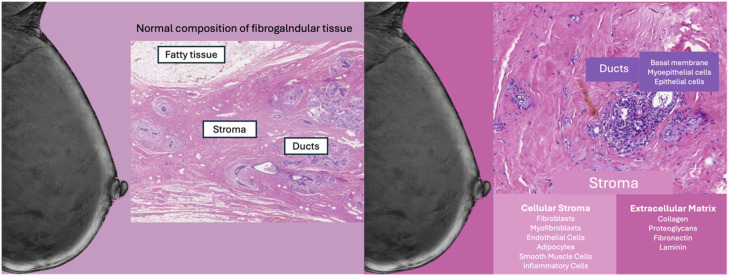
Breast tissue microscopic composition.

The usual composition of breast tissue consists of adipose tissue, stroma, and ducts. The stroma is composed of cellular stroma and extracellular matrix, as illustrated in Fig. 2. Currently, there is a lack of consensus on breast density in pathological anatomy, impeding comparability with radiological classification systems.

Limited publications address the correlation between breast tissue in pathological anatomy and mammographic imaging. One of those, an American cohort of 3400 individuals with benign breast diseases, is interesting^13^ as dense breasts were linked to two histological findings: fibrosis and absence of lobular involution. This conclusion concerning fibrosis aligns with other prior investigations that have additionally demonstrated the relationship^14^, ^15^, ^16^ Furthermore, type 1 lobules were correlated with heightened mammographic density (*p* = 0.021)^16^

Lobular involution is a typical aspect of breast aging, marked by a decrease in both the quantity and size of terminal duct lobular units and their secretory components, the acini. Lobular involution is frequently referred to as “physiological breast atrophy”. In four recent studies, the metric system of measurement was employed to standardize the evaluation of diseased breast density. Three papers constitute the Mayo Clinic Benign Disease Cohort in the United States, yielding noteworthy findings. The initial analysis, adjusted for age, parity, BMI, and menopause, indicated that women lacking lobular involution (OR = 1.7, 95 % CI 1.2‒2.3) and those with partial involution (OR = 1.3, 95 % CI 1.0‒1.6) exhibited a higher likelihood of possessing elevated DY mammographic density compared to those with complete lobular involution (*p* < 0.01)^17^ The second analysis indicated a significant connection between mammographically dense breasts and the absence of lobular involution in relation to breast cancer risk (OR = 4.08; 95 % CI 1.72 to 9.65; *p* = 0.006)^18^ In the third investigation, published by the same group in 2017^13^, of the 3400 individuals analyzed, 2163 (64 %) exhibited thick breasts. In a multivariate analysis, the lack of lobular involution (OR = 1.6; 95 % CI 1.2‒2.1) and fibrosis (OR = 2.2; 95 % CI 1.9‒2.6) demonstrated a statistically significant correlation with elevated mammographic density.

In an autopsy study involving 236 women, Li et al. conducted subcutaneous mastectomy during the autopsy, randomly selecting breast tissues to be analyzed using mammography. A high percentage of mammographic density was correlated with considerably increased total nuclear area, epithelial cell nuclear area, non-epithelial cell nuclear area, collagen proportion, and glandular structures (*p* < 0.001). Patients were categorized by age, and those aged 50-years or younger exhibited comparable relationships. For individuals over 50-years of age, only a subset of non-epithelial cell nuclear area and collagen had a significant correlation with dense breasts on mammography (*p* < 0.01 and *p* < 0.001, respectively)^19^

Ghosh et al. enrolled 59 symptomatic healthy volunteers with no previous breast cancer or hormone replacement therapy to conduct biopsies in both dense and non-dense regions of breast tissue guided by mammography and ultrasonography. Dense breast areas showed 4.8 % more epithelial content, 46.1 % increased stromal volume, and 50.9 % reduced adipose tissue compared to non-dense areas (all *p* < 0.0001). The proportion of lobular involution was greater in non-dense areas (73.9 %) compared to dense areas (52.2 %), albeit in a statistically non-significant manner.

### Breast density and cancer risk

Mammographic density is known to be a risk factor for breast cancer, associated with a 1- to 6-fold increase in the incidence of this disease^1^^,^^20^ The wide variability in risk might be linked to the different systems of classification employed throughout time^21^^,^^22^, and the transition from film mammography to digital mammography^23^, ^24^, ^25^

Individuals with extremely dense breast tissue (BI-RADS density D) had a 2.11-fold (95 % CI 1.84–2.42) higher risk of developing breast cancer than those with scattered dense breast tissue (BI-RADS density B), according to a meta-analysis that was published in 2022. The meta-analysis included nine observational studies. According to the sensitivity analysis, the probability of breast cancer increased by 1.83 times (95 % CI 1.52–2.21) when the data were corrected for age and body mass index^26^

In the past few years, the impact of breast density on the risk of breast cancer has been of such importance that it has been incorporated into epidemiologically based cancer risk calculation models, such as the Tyrer-Cuzick model (version 8), since 2022. This inclusion has resulted in superior modeling performance. The accuracy of risk stratification was enhanced by the inclusion of breast density in the Tyrer-Cuzick model. This is true for both high-risk (> 8 %, 10-year risk) and low-risk (< 2 %, 10-year risk) women. The proportion of patients classified as high risk increased from 4.8 % (no density) to 7.1 % (BI-RADS density) and 6.8 % (volumetric percent density)^27^^,^^28^

### How does breast density influence primary breast cancer screening?

As previously mentioned in this article, breast density raises the risk of breast cancer. Additionally, women with dense breasts have decreased screening mammography sensitivity due to the masking effect of dense (fibroglandular) breast tissue^1^^,^^29^ It was expected that when Digital Mammography (DM) replaced Film-Screen Mammography (FSM), screening performance would increase in women with dense breasts. Regretfully, when DM was utilized, screening sensitivity was considerably lower in women with dense breasts than in those non dense^30^^,^^31^

One study applied the volpara program for volumetric measurement of breast density from 2003 to 2011 to assess the detection and interval cancer rates of 111,898 mammograms from the biennial Dutch breast cancer screening program in women between the ages of 50 % and 75. 21.6 % of the breasts were found to be type A, 41.5 % to be type B, 28.9 % to be type C, and 8.0 % to be type D. 24-months following mammography, 234 interval cancers and 667 mammographically detected cancers were found. The higher density groups had reduced screening sensitivity: 85.7 %, 77.6 %, 69.5 %, and 61.0 % for groups A–D, respectively (*p* < 0.001). For categories A–D, the intervals for cancer detection/1000 exams were 0.7, 2.0, 3.1, and 4.3 (*p* < 0.001)^3^

### When it comes to dense breasts, what additional screening options are there?

The identification of breast cancer significantly impacts public health. Despite the growing number of other breast imaging modalities, mammography remains the primary tool for breast cancer screening. In women at average risk, going to annual screening mammography at age 40 has demonstrated a reduction in breast cancer mortality by roughly 20 % in long-term follow-up studies from randomized trials^32^^,^^33^ For women at intermediate and high risk, as well as those with dense breast tissue, supplementary modalities such as magnetic resonance imaging, ultrasound, tomosynthesis, and molecular breast imaging may be considered for enhanced screening to increase detection rates^34^

### How to categorize patient risk to tailor the screening for breast cancer?

To assess breast cancer risk for the recommendation of additional screening tests, a combination of criteria and evaluative tools is generally utilized, aiming to enhance early detection of aggressive breast cancers prior to their progression to advanced stages, while reducing screening-related harms. Basically, patients are categorized into average, intermediate, and high-risk classifications for breast cancer for developed invasive cancer. There are many breast cancer risk calculation, this group use the risk assessment instruments by the Breast Cancer Surveillance Consortium (BCSC) ‒ BCSC invasive breast cancer risk Calculator v3 FREE iPhone & Android (http://www.bcsc-research.org/data/index.html) due to provide more accurate risk estimation for women from diverse racial and ethnic^35^ and the BRCA Pro for women with BRCA1 and BRCA2 mutations. A woman at average-risk patient are almost 69 % of the population and is defined as one without also a personal history of breast cancer, a prior diagnosis of high-risk breast lesions such as Atypical Ductal Hyperplasia (ADH) or Lobular Carcinoma In Situ (LCIS), identified genetic mutations that increase breast cancer risk, or a childhood history of chest radiation exposure this group do not have beneficial in add additional exams for screening. Almost 12 % as defined as intermediate risk and 17 % as defined as high-risk patients according to the BCSC invasive breast cancer risk Calculator and may benefit from additional exams.

If assessing exclusively dense breasts, it is observed that 47 % of women have dense breasts (heterogeneously or extremely dense). Of these, 50 % of women with dense breasts are classified as low to average risk, whereas 24 % are at high risk of undetected cancer, and 27 % are at high risk of advanced cancer^36^^,^^37^

### Tomosyntheses

**Fig. 3:**
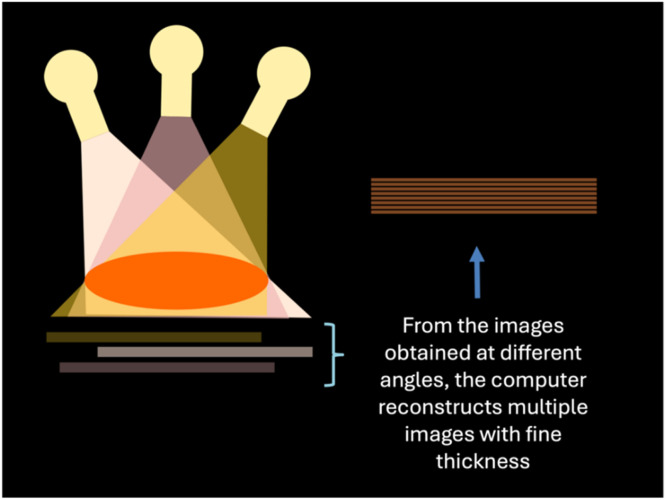
The process of image acquisition in tomosynthesis.

Digital Breast Tomosynthesis (DBT), also known as 3D mammography, acquires multiple low-dose X-Ray projections from different angles across the breast, which are reconstructed into thin slices (1 mm). DBT is especially valuable in heterogeneously dense breasts, where masking is common due to reduced tissue overlap and improves lesion visibility^38^ (Fig. 3).

Large prospective trials have demonstrated that the addition of DBT to DM increases cancer detection rates. The STORM and Oslo trials reported 2.3 to 2.7 additional cancers detected per 1000 screens when DBT was added to DM^39^^,^^40^ Friedewald et al. showed a 1.2 per 1000 increase (95 % CI 0.8–1.6) in a multicenter study using historical controls^41^

**Fig. 4:**
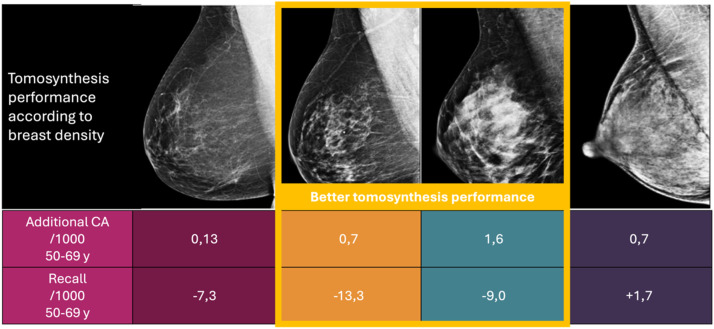
Tomosynthesis recall rates and performance.

A population-based cohort study involving 1.5 million screening exams (1.2 million with DM and 310,000 with DBT) showed that DBT significantly improved cancer detection across all age groups. In women aged 50–59, detection increased from 5.9 (DM) to 8.8 (DBT) per 1000 exams (RR = 1.50; 95 % CI 1.10–2.08). Similar trends were observed in other age groups: ages 40–49: 2.5 vs. 3.1 (RR = 1.28; 95 % CI 1.02–1.61); ages 50–59: 3.7 vs. 5.3 (RR = 1.42; 95 % CI 1.23–1.64); ages 60–79: 6.1→8.5 (RR = 1.39; 95 % CI not specified)^5^ In heterogeneously dense breasts, DBT significantly improved invasive cancer detection across all age ranges. Among women with scattered fibroglandular density, DBT increased overall detection in the 50–59 age group 3.3 to 4.0 per 1000 (RR = 1.21; 95 % CI 1.02–1.42), and in the 60–79 age group from 5.5 to 6.5 per 1000 (RR = 1.18; 95 % CI 1.03–1.35). For invasive cancers in women aged 50–59, detection increased from 2.4 to 3.0 per 1000 (RR = 1.23; 95 % CI 1.02–1.48). However, in women with extremely dense or almost entirely fatty breasts, the overall cancer detection rates were similar between DM and DBT, suggesting that DBT’s benefit may be more limited in those subgroups^42^ (Fig. 4).

Overall, DBT offers a meaningful improvement in screening performance, especially for women with heterogeneously dense tissue, while also reducing recall rates. These features support its integration into population-based breast cancer screening programs.

### Contrast-enhanced spectral mammography

Contrast-enhanced spectral mammography, also known as CEM, has recently emerged as a potentially useful alternative to breast Magnetic Resonance Imaging (MRI), particularly in the evaluation of women who have dense breasts^43^ By combining contrast-enhanced recombined views with low-energy mammographic images, Contrast-Enhanced recombined Mammography (CESM) provides functional information that is comparable to that of Magnetic Resonance Imaging (MRI), but with more accessibility, shorter acquisition time, and a lower cost. Recent research has shown that CESM is capable of achieving a pooled sensitivity of 97 % and a specificity ranging from 66 % to 84 % in the identification of breast cancer in women who have dense breasts. These values are comparable to or approaching those of MRI^44^^,^^45^ CESM has demonstrated advantages over digital mammography and tomosynthesis in dense breast tissue over the course of supplemental screening, with greater cancer detection rates and fewer false negatives^46^ This is the case in the context of supplemental screening. Furthermore, CESM has shown clinical utility in preoperative staging, problem-solving for ambiguous lesions, and post-treatment surveillance. It frequently matches or roughly approximates the performance of MRI, particularly in situations where MRI is either not accessible or is contraindicated^44^^,^^47^ Ongoing prospective trials, such as the CMIST research, are being conducted with the purpose of further validating CESM as a viable and efficient option for breast cancer screening and diagnosis in women who have dense breasts^48^

### Breast ultrasonography

Regarding the screening for breast cancer, there is no consensus with respect to whether an ultrasound of the breast is essential. In this scenario, ultrasonography gained popularity following the American effort to decrease false negatives in mammography, which are primarily associated with dense breasts, and there is a movement to personalize screening.

Three significant studies investigated the efficacy of combined mammography and ultrasonography for breast cancer screening. All three demonstrated a decrease in interval cancer and an increase in breast cancer detection rates and sensitivity^49^, ^50^, ^51^

The Japanese study was the only randomized trial involving 11,390 participants at risk. Complementary screening with ultrasound improved sensitivity from 70.6 % to 93.2 %, enhanced the detection rate to 1.8 per 1000 exams, and reduced interval cancer incidence to 0.5 per 1000 exams, instead of 2 per 1000 exams in the mammography group^49^

Two additional cohort studies involving around 1600 and 1100 asymptomatic patients with heterogeneously and extremely dense breasts indicated that the incorporation of ultrasound into screening increased the cancer detection rate from 4.2 to 6.6 per 1000 women screened[52] and from 3.4 to 6.8 per 1000 women^51^

Complementary ultrasound screening has the potential to identify cancers missed by mammography; however, this advantage may be mitigated by potential drawbacks, including elevated recall rates for additional imaging, which can range from 8.6 % to 26.6 % in the mentioned studies, as well as increased rates of additional breast biopsies^49^, ^50^, ^51^

The proper balance of advantages and disadvantages may be improved by focusing ultrasound screening on women at elevated risk of mammography screening failure. Besides breast density, several breast cancer risk factors ‒ such as familial history of breast cancer, prior diagnosis of benign breast disease, and obesity ‒ have been demonstrated to correlate with the likelihood of mammography screening failure^52^^,^^53^

### Breast magnetic resonance imaging (MRI)

The role of Magnetic Resonance Imaging (MRI) in breast cancer screening has gained increasing recognition, particularly for women with dense breast tissue. Several prospective multicenter trials have demonstrated the superior diagnostic performance of MRI compared to X-Ray-based modalities, including digital mammography and tomosynthesis, as evidenced by higher cancer detection rates and a reduction in interval cancers^54^, ^55^, ^56^

The largest randomized study comparing MRI and mammography is the DENSE trial, conducted in the Netherlands, which enrolled 40,373 women with extremely dense breasts. The interval cancer rate was 2.5/1000 exams in the MRI group and 5.0/1000 in the mammography group. MRI also detected 16.5 cancers per 1000 screening exams, demonstrating significantly higher sensitivity^55^ After two years and a second screening round, the rate of cancers detected by mammography in the MRI group decreased to 5.8 per 1000, suggesting a sustained screening effect^56^ These findings are supported by another prospective study involving 1400 participants, which found 11.8 invasive cancers per 1000 women screened with MRI, versus 4.8 per 1000 with mammography^54^

Despite its advantages, MRI is associated with increased recall and biopsy rates. A meta-analysis from a Canadian group showed that the recall rate rose from 3 % to 9.5 %, and biopsy rates from 3 % to 6 %, following MRI screening. Importantly, false positives accounted for 82 % of all recalls. Nonetheless, the rate of adverse events was low, estimated at approximately 0.1 %^53^ A notable advantage of MRI is the absence of ionizing radiation, although contraindications such as claustrophobia and metal implants may limit its use.

MRI has also demonstrated superior performance compared to tomosynthesis. In a study of over 1440 at-risk women, MRI detected 15.2 cancers per 1000, while tomosynthesis detected only 6.2 per 1000^54^

The BRAID trial is the first large-scale randomized study to directly compare three supplemental imaging techniques ‒ abbreviated breast MRI (abMRI), Contrast-Enhanced Mammography (CEM), and automated breast ultrasound (ABUS) **‒** in women with dense breasts and negative screening mammograms, published in 2025. Both abMRI and CEM demonstrated substantially higher cancer detection rates (17.4 and 19.2 per 1000, respectively) compared to ABUS (4.2 per 1000), with most tumors detected being small, node-negative, and invasive, suggesting effective early detection. The median tumor size was smallest with abMRI (10 mm), followed closely by CEM (11 mm), whereas ABUS-detected tumors were larger (22 mm), and no cases of DCIS were identified with ABUS. Although recall and biopsy rates were higher with abMRI and CEM (∼9.7 % recalls; ∼4.5 % biopsies), the Positive Predictive Value (PPV) for biopsies was also greater ‒ 43.8 % for CEM and 35.2 % for abMRI versus 28.1 % for ABUS. Overall, CEM and abMRI performed similarly, offering a balance between sensitivity and diagnostic value, while ABUS showed limited performance in this context, despite a lower rate of false positives. These findings support the potential use of contrast-enhanced modalities as effective supplemental tools in women with dense breasts, though considerations regarding contrast risks, cost, and availability remain crucial for implementation^57^

Currently, annual MRI screening is recommended for women at high risk of breast cancer, as defined by the Breast Cancer Surveillance Consortium (BCSC) risk calculator (http://www.bcsc-research.org/data/index.html)^35^ In 2022, the European Society of Breast Imaging (EUSOBI) expanded MRI recommendations to include women with extremely dense breasts, advising screening at least every four years, with a preferred interval of two to three years^58^

### Innovations in technology

The breast parenchyma exhibits textural features that can function as imaging indicators to detect alterations associated with breast cancer, in addition to playing a role in the categorization of malignancy subtypes^59^^,^^60^ Evidence suggests that these features represent biological risk factors that may affect cancer development, hence enhancing the precision of individual risk assessment in women^61^^,^^62^ In 2016, Gastounioti et al. categorized methodologies for breast parenchyma texture into five different classifications. They determined that multiparametric characteristics are superior in predicting breast cancer compared to unidimensional characteristics^63^

A systematic evaluation of 28 studies that evaluated the usefulness of textural features in predicting breast cancer was published in 2022. The findings show that when texture metrics are added to mammographic density, risk prediction models are improved. For more reliable results, data with longer intervals between mammograms and diagnoses is required, as most studies assessed a time frame of less than three years. Furthermore, consistent texture evaluation is necessary to carry out effective cross-study comparisons^64^ Consequently, examinations of breast parenchymal texture in the assessment of breast cancer risk have increasingly acquired importance.

Artificial Intelligence (AI) has revolutionized breast cancer risk evaluation by enhancing the precision of screening and diagnosis. It analyzes mammographic pictures to detect risk-associated attributes, including breast density, mass characteristics, texture, and location. Moreover, AI can use variables such as age, familial history, and genetic predispositions to generate individualized risk assessments, demonstrating significant promise in breast cancer screening^65^, ^66^, ^67^

Data-driven techniques, especially Deep Learning (DL) and Convolutional Neural Networks (CNNs), are commonly utilized in Artificial Intelligence (AI). Convolutional Neural Networks (CNNs), a subset of Deep Learning (DL), handle image-related tasks such as detection, segmentation, and classification, and may assess breast cancer risk from mammograms, either independently or alongside additional criteria^68^, ^69^, ^70^

Nonetheless, the effective incorporation of AI into healthcare workflows and transparent communication between patients and clinicians are essential for its proper utilization. Although AI presents great opportunities for breast cancer prevention and early detection, it is crucial to address technical, ethical, and practical challenges through additional research to facilitate its broader clinical application^71^

Traditional clinical algorithms, including Gail/BCRAT, BCSC, and Tyrer-Cuzick, show varying results, with AUC values ranging from 0.57 to 0.82^72^ Recent findings suggest that AI-driven models that combine traditional risk factors with mammographic pictures can markedly enhance the efficacy of epidemiological models. AI methodologies utilized on datasets from various medical institutions have indicated C-index values between 0.75 and 0.84^73^, demonstrating significant potential improvements in risk evaluation.

Recent research indicates that Liquid Biopsy (LB) may provide a significant non-invasive method for the early diagnosis of breast cancer, especially in populations facing diagnostic difficulties, such as women with dense breast tissue. LB comprises various circulating biomarkers derived from blood, urine, or saliva, such as cell-free DNA (cfDNA), circulating tumor DNA (ctDNA), Circulating Tumor Cells (CTCs), microRNAs (miRNAs), long non-coding RNAs (lncRNAs), Tumor-Educated Platelets (TEPs), and Volatile Organic Compounds (VOCs). A number of studies have shown encouraging diagnostic accuracy for these biomarkers, with sensitivities reaching 98.6 % and specificities nearing 100 % for specific miRNA and ctDNA signatures^74^, ^75^, ^76^ Circulating free DNA (cfDNA) and circulating tumor DNA (ctDNA) have demonstrated the capability to identify significant mutations and methylation patterns linked to cancer, including PIK3CA, EGFR, and TP53. Exosomal lncRNAs, urine VOCs^40^, and salivary mRNAs and proteins^77^^,^^78^ have produced promising outcomes in limited investigations. Notwithstanding these advancements, many limitations persist, including inconsistency in detection methodologies, reduced sensitivity in early-stage disease for certain markers, and the absence of standardization. Nevertheless, incorporating liquid biopsy into breast cancer screening protocols ‒ particularly for high-risk populations ‒ could improve early detection and facilitate more tailored monitoring measures, supplementing conventional imaging methods.

### Legal implication

In 2004, Dr. Nancy Capello established the group “Are You Dense” in the United States to provide legal assistance to individuals with dense breast tissue, advocating for supplementary screening after her diagnosis of metastatic breast cancer despite a normal mammogram. Subsequently, some American states have implemented regulations to notify patients about breast density and the potential need for further diagnostic testing, due to the increased cancer risk linked to dense breasts. Shared decision-making between physicians and patients is gaining popularity, emphasizing the necessity of accurately categorizing breast density to identify candidates for supplementary screening, while considering cost-effectiveness and the safety of patients, as advised by the American College of Radiology^79^

### Recommendations from medical societies on screening for dense breasts

Houssami’s 2023 meta-review assessed 23 international breast cancer screening guidelines released from 2014 to 2024, focusing on women with mammographically dense breasts. The guidelines from North America, Europe, Asia, and Oceania show significant variability in methodological quality, with only 10 obtaining a score above 60 % on the AGREEII scale. Many performed in clarity but received low scores in application, providing minimal assistance for real-world implementation. Mammography was consistently advocated as the main screening method, however, recommendations for additional imaging varied. Approximately one-third of the guidelines recommended against additional imaging, although others endorsed adjunct modalities including ultrasound (7 guidelines), MRI (3), Digital Breast Tomosynthesis (DBT) (3), and Contrast-Enhanced Spectral Mammography (CESM) (2). These discrepancies indicated a constrained evidence foundation, frequently based on observational data or expert consensus. Moreover, the inconsistency in evidence grading systems led to interpretation diversity among guidelines^80^

The lack of breast density and breast cancer screening legislation in Brazil presents complex barriers for clinical practice. Diverse medical associations present contradictory recommendations, potentially resulting in confusion among doctors and inconsistencies in patient management. Health management organizations, which oversee and facilitate health services, and implementation societies, which provide clinical care, have different criteria. The Brazilian College of Radiology (CBR), the Brazilian Society of Breast Imaging (SBM), and the American College of Radiology (ACR) advocate for supplementary ultrasound in women with dense breasts, highlighting the inadequacies of mammography in this demographic. The National Cancer Institute (INCA) and European recommendations do not endorse ultrasound as a regular supplementary technique, prompting continuous discussions about its efficacy and cost-effectiveness.

**Fig. 5:**
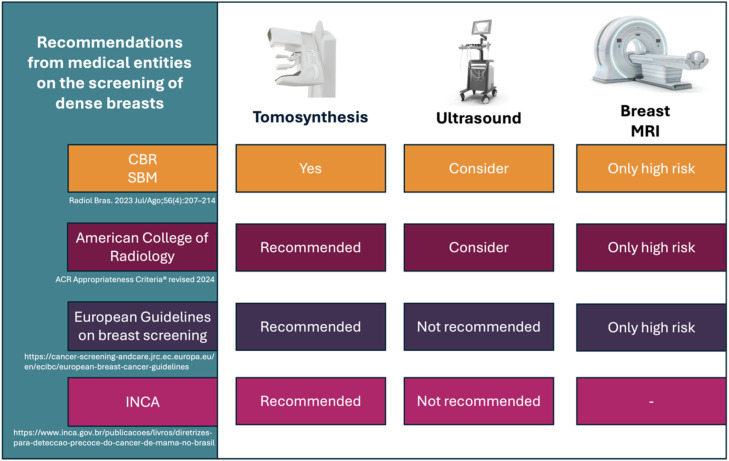
Recommendations from medical entities on the screening for dense breast.

**Fig. 6:**
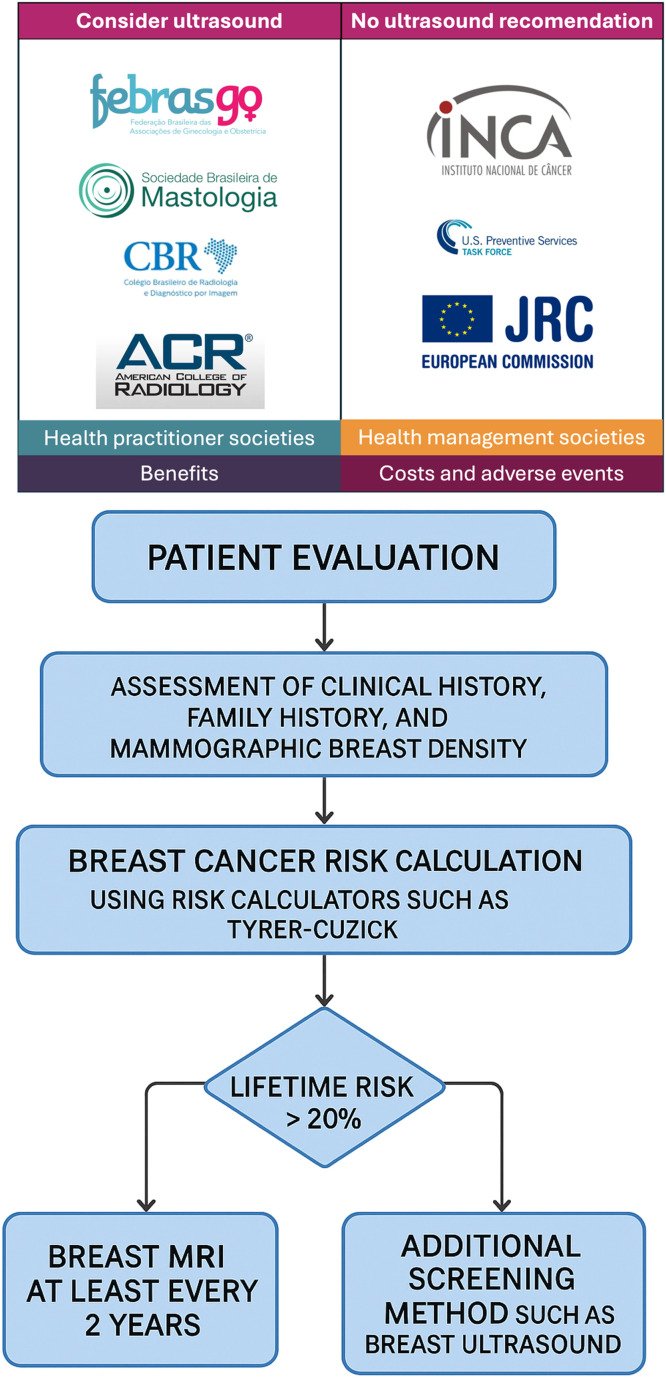
(6.1) Examples of different recommendations for ultrasound screening between health practitioner societies and health management societies and (6.2) figure on how to perform in clinical practice.

There is a general agreement that breast MRI should not be employed routinely for dense breast tissue. Current clinical guidelines restrict MRI utilization to women with a lifetime breast cancer risk beyond 20 %, highlighting the significance of personalized risk evaluation. This highlights the necessity for explicit, evidence-based national recommendations that standardize screening techniques and diminish variability in clinical decision-making, especially within a resource-limited and inequitable healthcare system like that of Brazil. Figs. 5 and 6 encapsulate the guidelines for medical organizations, health managers, and practitioners.

### Limited from current evidence and global disparities

Study data on dense breasts indicates considerable variations in the definition, evaluation, and incorporation of breast density into screening protocols. While most guidelines incorporate the BI-RADS classification, most fail to delineate the measuring methodology or utilize objective equipment, hence constraining comparability between research and undermining therapeutic standardization^81^

Guidelines also vary in their approach to dense breast tissue. North American guidelines, including those from the USPSTF, American College of Radiology, and American Cancer Society, often advocate for a conservative and individualized approach, highlighting the inadequate evidence to support widespread supplemental screening. Recognizing the low sensitivity of mammography in dense breast tissue, they often endorse a collaborative approach to decisions for supplementary imaging (such as MRI or ultrasound), focused on individual risk factors [88]

However, EUSOBI and European Commission guidelines actively recommend Magnetic Resonance Imaging (MRI) for women with dense breasts, supported by research like the dense study.

Guidelines in Low- and Middle-Income Countries (LMICs) such as Brazil, China, and India are often more cautious. The Brazilian guidelines, developed by FEBRASGO, the Brazilian College of Radiology (CBR), and the Brazilian Society of Mastology (SBM), endorse annual digital mammography starting at age 40 and suggest adjunctive ultrasonography for women with dense breast tissue. However, it does not specify objective criteria for indication, frequency, or cost-effectiveness of additional imaging, highlighting a situation where access to advanced technology is limited. This landscape highlights scientific limitations as well as socioeconomic and equity challenges. The overwhelming majority of current evidence derives from high-income countries, predominantly including White populations with reliable access to expensive imaging technology and organized screening protocols. Implementing these principles in Brazil requires caution, taking into account barriers to diagnostic imaging, geographical disparities, and limited access to MRI and tomosynthesis within the public healthcare system (SUS).

Furthermore, the implementation of strategies such as mandatory breast density notification, established in certain U.S. states, raises ethical and practical challenges. In situations lacking fair access to supplementary imaging or well-defined treatment routes, notifying patients about thick breasts without providing feasible options may result in worry, confusion, and exacerbation of health disparities, rather than promoting autonomy.

Consequently, guidelines for managing dense breasts must take into account not only the scientific evidence but also the logistical, economic, and social dimensions of their execution. Brazil and other LMICs must carefully adapt international recommendations to their local contexts, integrating cost-effectiveness assessments as well as strategies for supplemental screening that address health disparities while ensuring universal, continuous, and effective access to early breast cancer detection.

### Future directions

Breast cancer screening is set to advance beyond the conventional age-based, standardized methods that have characterized practice for decades. Recent advancements in genomics and medical technology are instigating a paradigm shift in breast cancer screening, transitioning from age-based protocols to multivariate, risk-adapted models focused on individual risk profiles.

A promising turning point is Polygenic Risk Scores (PRS), which quantify inherited susceptibility by aggregating the effects of many common genetic variations that contribute moderately to disease risk. In oncology, particularly breast cancer, PRS can identify individuals at significantly elevated lifetime risk, usually comparable to or exceeding that of carriers of moderate-penetrance mutations like CHEK2 or PALB2, even without a family history.

Future screening frameworks are therefore expected to incorporate PRS alongside parameters such as breast density, imaging biomarkers, and reproductive history to enhance the precision of risk stratification. The continuous distribution of PRS within the population enables a nuanced approach to screening decisions, as opposed to binary classifications. Despite current implementation challenges ‒ including ancestry-related biases, lack of standardization in score construction, and complexities in risk communication ‒ PRS holds significant promise for enhancing the effectiveness, efficiency, and equity of breast cancer prevention. Realizing this potential will require interdisciplinary collaboration among clinicians, geneticists, public health experts, and patient advocacy groups to ensure that these advances are translated into ethically sound and contextually appropriate clinical practice within personalized and participatory healthcare frameworks.

A large-scale population-based investigation by Mars et al. in the FinnGen cohort showed that PRS can stratify breast cancer risk. Over 117,000 women were analyzed to assess the predictive power of high PRS (above the 90th percentile) with family history and Pathogenic Variants (PVs) in CHEK2 and PALB2. High PRS was related to a breast cancer HR of 2.3 during the target screening age (50–69 years), equivalent to PVs or positive family history. These data show that PRS can be used to customize screening onset, intensity, and modality and improve predictive accuracy when combined with other hereditary risk indicators. Mars et al. recommend assessing the clinical implementation and cost-effectiveness of stratified screening methods in real-world health systems [90]

Actually, two large international randomized clinical trials ‒ WISDOM in the US and MyPeBS (My Personal Breast Screening) in the EU ‒ are validating and implementing personalized breast cancer screening. In women 40–70, MyPeBS compares age-based screening to risk-stratified screening. The personalized arm assigns screening frequency and modality based on the individual's estimated 5-year risk of invasive breast cancer, calculated using clinical and reproductive history, breast density, lifestyle factors, and a Polygenic Risk Score (PRS313) derived from saliva-based genotyping of 313 validated polymorphism [91]

It is evident that a significant disparity exists between the generation of scientific evidence, policy development, and clinical implementation, particularly in nations with little purchasing power. The majority of existing research focuses on Caucasian populations characterized by high educational attainment and consistent access to new technologies, hence restricting their direct relevance to the Brazilian environment. Consequently, there is a crucial requirement for national studies in Brazil to evaluate:

• The prevalence and distribution of breast density among various Brazilian populations.
• The efficacy and cost-effectiveness of supplementary screening modalities (ultrasound, tomosynthesis, magnetic resonance imaging).
• The influence of these measures on pertinent clinical outcomes, including early detection, diagnostic timelines, and the decrease in advanced-stage malignancies.
• And the equity of access and outcomes, considering geographical, racial, and socioeconomic differences.

This information is crucial for establishing therapeutic guidelines that combine scientific rigor, practical feasibility, and an effective commitment to mitigating health disparities.

## Declaration of competing interest

The authors declare no conflicts of interest.
